# Cultural Adaptation of Visual Attention: Calibration of the Oculomotor Control System in Accordance with Cultural Scenes

**DOI:** 10.1371/journal.pone.0050282

**Published:** 2012-11-19

**Authors:** Yoshiyuki Ueda, Asuka Komiya

**Affiliations:** 1 Kokoro Research Center, Kyoto University, Kyoto, Japan; 2 Graduate School of Humanities, Kobe University, Kobe, Japan; Ecole Normale Supérieure, France

## Abstract

Previous studies have found that Westerners are more likely than East Asians to attend to central objects (i.e., analytic attention), whereas East Asians are more likely than Westerners to focus on background objects or context (i.e., holistic attention). Recently, it has been proposed that the physical environment of a given culture influences the cultural form of scene cognition, although the underlying mechanism is yet unclear. This study examined whether the physical environment influences oculomotor control. Participants saw culturally neutral stimuli (e.g., a dog in a park) as a baseline, followed by Japanese or United States scenes, and finally culturally neutral stimuli again. The results showed that participants primed with Japanese scenes were more likely to move their eyes within a broader area and they were less likely to fixate on central objects compared with the baseline, whereas there were no significant differences in the eye movements of participants primed with American scenes. These results suggest that culturally specific patterns in eye movements are partly caused by the physical environment.

## Introduction

A vast amount of literature has documented cultural differences in cognitive processing. East Asians such as the Chinese and Japanese are likely to holistically recognize an object, taking contextual information into account or judging a target from a relational perspective. On the other hand, Westerners, especially people from the United States, are more likely than East Asians to be analytical, tending to ignore contextual information to focus on the central object or to judge a target from a categorical perspective (e.g., [1]–[10]). Although most cultural psychologists place emphasis on aspects of the social environment such as religion [11] or self-construals [12] to explain the origin of such differences, Miyamoto, Nisbett, and Masuda [13] indicated another possibility: the physical environment of a given culture. Specifically, because the Japanese live in a visually complex environment in which objects frequently overlap and have borders that are too ambiguous for people to distinguish them from the background, the Japanese may have a greater tendency than the Americans do to distribute their attention and direct it to the background. Consistent with their prediction, these authors showed that Japanese scenes are more complex and ambiguous than American scenes are and, more importantly, that participants who were continuously exposed to Japanese scenes were more likely to detect changes in contextual information than were participants who were continuously exposed to American scenes irrespective of their culture of origin.

Although the findings of Miyamoto et al. [13] suggest that absorbing one’s physical environment influences holistic/analytic cognitive processing, the study did not directly examine patterns of visual attention, that is, how people perceive cultural scenes. In this sense therefore, the link between people’s perception of cultural scenes and their attention toward these scenes is missing. One explanation may be that seeing a cultural environment, especially complex Japanese scenes, prompts the eye to scan contextual information. If this is the case, particular cultural environments should generate common attentional patterns.

Although many studies have confirmed the existence of cultural differences in a relatively high-order cognitive system, whether cultural differences exist in processing of a more fundamental nature, such as oculomotor control [1], [14]–[19], is still debatable. Chua et al. [1] displayed pictures of focal, foregrounded objects with realistic complex backgrounds while recording participants’ eye movements, and asked the participants to report their preferences. They found that American participants were more likely than Chinese participants to ignore the backgrounds and move their eyes toward a single object and fixate on it. However, other studies [17]–[19] did not show such differences: both American and Chinese participants in these studies were more likely to focus on focal objects than the background. Most strikingly, Evans et al. [17] used the same paradigm and stimulus as Chua et al. [1] did, but they could not replicate Chua et al.’s findings.

These mixed results might be partly due to the fact that the aforementioned researchers generally focused on differences in language and ignored participants’ visual experiences. In the above studies [1], [17]–[19], the culture to which a participant belonged was determined on the basis of the language he or she used in daily life. In these studies, American and Chinese participants were graduates or undergraduates at U.S. universities–“Chinese participants” were persons who were born in Chinese families and who could speak and read Chinese, but they were living in the United States at the time they participated in the experiments. Therefore, their visual experiences of their cultural scenes–how long, how likely, and how recently they were exposed to Chinese scenes–was not given much importance in the previous studies. If oculomotor control is calibrated by the cultural scenes that one is exposed to, not language, the differences in previous results might be attributable to the diversity of such visual experiences with cultural scenes. Hence, in the present study, we presented typical cultural scenes to participants who belonged to the same culture and could speak and read the same language, and investigated change in patterns of eye movements in response to natural scenes.

The purpose of the present study is to cohere the diverse possibilities afforded by physical environments and oculomotor control by investigating patterns of eye movements. If visual attention is fine-tuned by the visual environment, oculomotor control may be calibrated according to cultural scenes. Thus, repeated exposure to a cultural scene shapes in turn a specific style of attention control, such as holistic or analytic attention. In the experiment, we recorded eye movements of participants before, during, and after their observation of typical Japanese and American scenes and investigated changes in their oculomotor patterns. We predicted that patterns of eye movements would be more distributed during observation of Japanese scenes than they would during observation of American scenes. Moreover, after adaptation, patterns of eye movements would persist even when they were seeing “culturally neutral” scenes; that is, participants who adapted their attention to Japanese environments would be more likely to diffuse their attention than would participants who adapted their attention to American environments.

Additionally, we investigated instances in which the patterns of participants’ eye movements were more influenced by their adaptation to cultural scenes. For this purpose, we introduced two factors into neutral scenes: scene structure (a single focal object vs. multiple distributed objects) and repetition of scenes (one-time presentation vs. repeated presentation). Boland, Chua, and Nisbett [20] suggested that Rayner et al. [18] could not replicate their results because the discrepancy between them was due to scene structure. They argued that Rayner et al.’s [18] use of multiple-objects scenes–instead of single-object scenes like those used by Chua et al. [1]–might not engender cultural differences. Moreover, we can investigate the effect of the top-down strategy concerned with viewing scenes by comparing patterns of eye movements during repeated presentations of the same scene with patterns during the presentation of a novel scene, because participants acquire a general understanding of a scene when it is presented repeatedly. Introducing these factors into our analysis enabled us to ascertain which traits of the scenes were influenced by adaptation.

## Methods

### Ethics Statement

The procedures was approved by the internal review board of Kyoto University, and written informed consent (study purpose, methodology, risks, the right to withdraw, duration of the experiment, dealing with individual information, and voluntariness) was obtained from all participants prior to the testing.

### Participants

A total of 32 Japanese undergraduates at Kyoto University, Japan participated in the experiment in return for 1,000 Japanese yen. All participants had normal or corrected-to-normal vision.

### Materials

The stimulus was presented on a CRT monitor (Dell UltraScan P991, 19 inch, 35 cm×26 cm) with 1,024×768-pixel resolution and a 60-Hz refresh rate. Stimulus presentation was controlled by Windows XP and MATLAB (Math Works) with Psychophysics and Eyelink Toolbox ([21]–[23]; http://psychtoolbox.org/). Participants watched the monitor with both eyes, and the movements of their left eyes were recorded via SR Eyelink 1000 (SR Research). The position of each participant’s head was fixed by a chin rest to maintain the distance between the head and the CRT at 75 cm.

**Figure 1:**
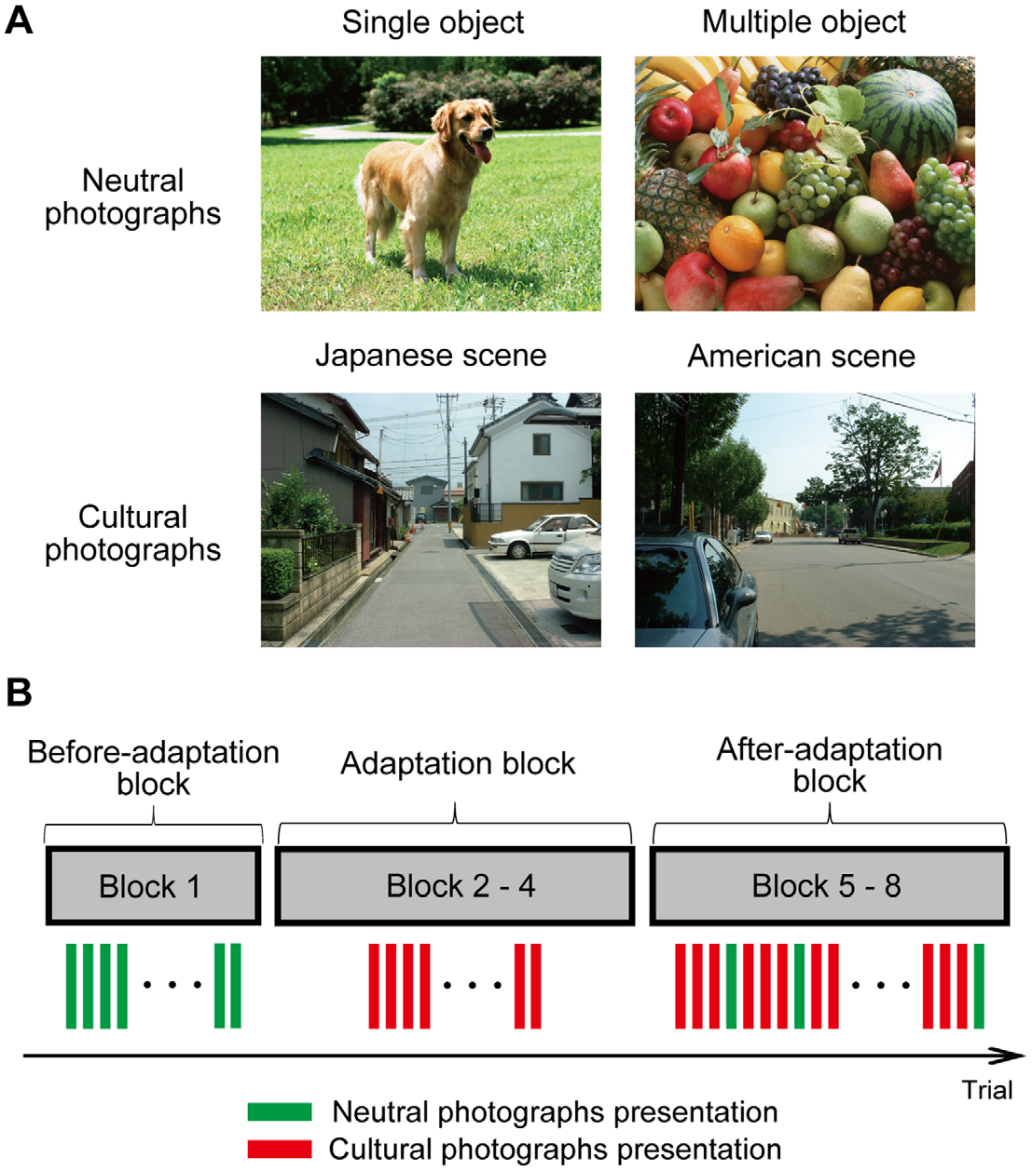
Materials used in this study. (A) Examples of photographs used in this study. Single-object (upper left) and multiple-objects photographs (upper right) were presented in the before- and after-adaptation blocks. Photographs of Japanese scenes (bottom left) and American scenes (bottom right) were presented in the adaptation and after-adaptation blocks. All photographs were presented in color. (B) The order of photograph presentation is shown.

We used four categories of photographs. As culturally neutral stimuli, we selected 30 single-object photographs and 30 multiple-objects photographs from sozaijiten.com. As typical cultural scene stimuli, 240 photographs of Japanese scenes (taken in Tokyo, Hikone, and Torahime) and 240 photographs of American scenes (taken in New York City, Ann Arbor, MI, and Chelsea, NY) were used; these were the same pictures used in the study by Miyamoto et al. [13]. Examples are shown in Figure 1a. All photographs were displayed in color, and their visual size was 26°×20°.

### Procedure

In each trial, the fixation cross was presented for 500 ms, randomly placed near either the left or right edge of the monitor, and accompanied by a beep. Following the fixation cross, one photograph was presented in the center of the monitor for 2,000 ms, during which participant’s eye movements were recorded. After the photograph disappeared, participants reported the degree to which they liked it (1: do not like at all; 7: like very much). No time limit was placed on participants’ reporting.

After signing an informed consent, participants were randomly assigned to either the Japanese- or American-scene condition. They performed 320 trials, divided into eight blocks of 40 trials, with a short break between blocks (Figure 1b). The first block was the *before-adaptation* block, in which 20 single-object and 20 multiple-objects photographs were presented to measure initial eye movement patterns. The second to fourth blocks were *adaptation* blocks, in which the participants assigned to the Japanese-scene condition saw 120 Japanese street photographs, and the participants assigned to the American-scene condition saw 120 photographs of American scenes. The fifth to eighth blocks were *after-adaptation* blocks, in which 20 single-object photographs and 20 multiple-objects photographs were presented every fourth trial, following three Japanese- or American-scene photographs. These photographs of cultural scenes were presented to prevent weakening of the adaptation. Half of the single-object and multiple-objects photographs in the after-adaptation block were presented in both the before- and after-adaptation blocks (repeated presentation), and the remainder were novel to the participants (one-time presentation). Photographs were randomly assigned to either the one-time or repeated presentation condition and presented in a random order in each block.

## Results

For our analyses, short fixations of less than 80 ms were excluded. This cutoff is often used because observers do not integrate visual information in this short period (e.g., [24]–[25]).

### Eye Movements during Observation of Cultural Scenes

**Figure 2:**
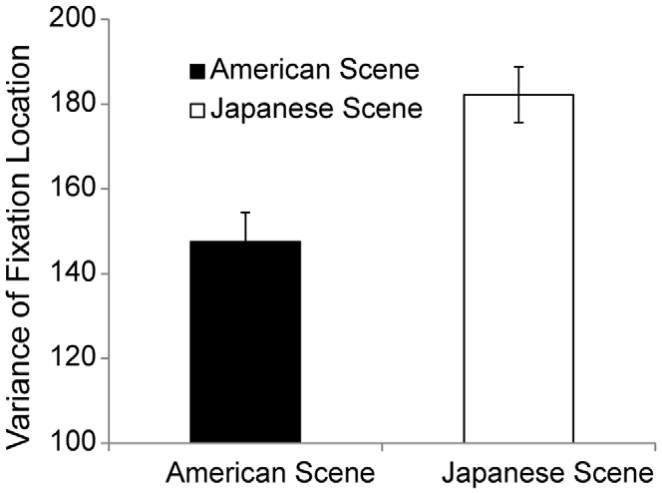
Mean variances in fixation during presentation of cultural scenes in the adaptation and after-adaptation blocks. Black bars indicate variances when participants saw Japanese scenes, and white bars indicate variances when participants saw American scenes. Error bars show standard errors.

First, to reveal the differences in eye movements between the Japanese and American scenes, we analyzed the patterns of eye movements in the adaptation blocks. For fixation deviation, the mean distance from the averaged location of all the observed fixations to each fixation location, indicating fixation distribution, in the adaptation blocks are shown in Figure 2. A *t*-test showed significant differences between the Japanese- and American-scene conditions (182.2 for the Japanese-scene condition and 147.6 for the American-scene condition), *t*(30) = 3.64, *p*<.005, *r* = .55, suggesting that participants moved their eyes more broadly when viewing Japanese than when viewing American scenes. The results were consistent with our predictions.

The fixation durations were slightly longer in the American-scene condition than the Japanese-scene conditions, but it was not statistically significant (285.4 ms for the Japanese-scene condition and 308.0 ms for the American-scene condition), *t*(30) = 1.58, *n.s*.

### Eye Movements after Observation of Cultural Scenes

**Figure 3:**
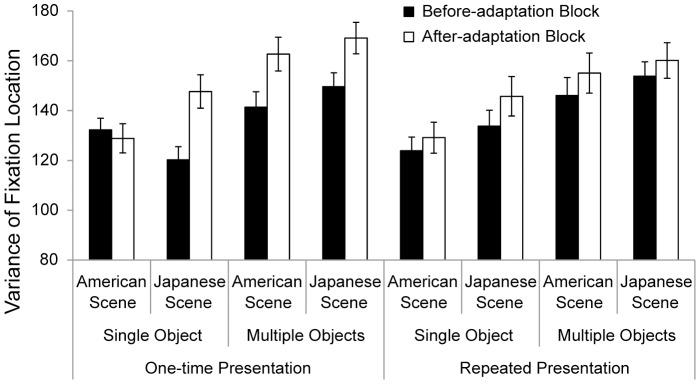
Mean variances in fixation during presentation of neutral scenes in the before- and after-adaptation blocks. Black bars indicate variances in the before-adaptation blocks, and white bars indicate variances in the after-adaptation blocks. Error bars show standard errors.

To examine whether participants’ eye movements changed between the before- and after-adaptation blocks, we compared variances in fixation between these blocks. The means and standard errors are shown in Figure 3. A 2 (scene: Japanese or American scene) × 2 (block: before or after adaptation) × 2 (object: single or multiple objects) × 2 (presentation: one-time or repeated) repeated-measures ANOVA on the mean variances in fixation showed a significant three-way interaction of scene × block × object × presentation, *F* (1, 30) = 4.74, *p*<.05, *ŋ_p_^2^* = .14. To assess the influence of the acquired knowledge of the scene, planned comparisons were conducted with respect to each presentation condition. In the one-time presentation condition, a two-way interaction of scene × block × object was significant, *F* (1, 30) = 10.39, *p*<.005, *ŋ_p_^2^* = .26. Follow-up analyses revealed the significant interaction of scene × block for the single-object, one-time presentation condition, *F* (1, 30) = 8.64, *p*<.01, *ŋ_p_^2^* = .22, showing that participants who saw Japanese scenes diffused their fixation more after the adaptation block than before (120.3 for the before adaptation block and 147.7 for the after adaptation block), *F* (1, 15) = 15.22, *p*<.005, *ŋ_p_^2^* = .50, whereas participants who saw American scenes did not (132.3 for the before adaptation block and 128.9 for the after adaptation block), *F* (1, 15)<1, *n.s*., *ŋ_p_^2^* = .01. Under the multiple-objects condition with one-time presentation, in contrast, only the main effect of block was significant, *F* (1, 30) = 21.26, *p*<.001, *ŋ_p_^2^* = .41, showing that for scenes contained multiple-objects, participants in both Japanese- and American-scene conditions were more likely to distribute their attention after the adaptation block than before that block (145.5 for the before adaptation block and 165.9 for the after adaptation block).

On the other hand, under repeatedly presentation, the main effect of object was significant, *F* (1, 30) = 27.78, *p*<.001, *ŋ_p_^2^* = .48, and the main effect of block was marginally significant, *F* (1, 30) = 3.88, *p*<.10, *ŋ_p_^2^* = .11, but there was no significant interaction of scene × block × object, *F* (1, 30)<1, *n.s.*, *ŋ_p_^2^* = .03. This suggests that participants’ fixations were more diffused under the multiple-objects condition and after the adaptation block than under the single-object condition or before the adaptation block. The results for the repeated-presentation condition were not surprising because acquired knowledge about the scene can move attention toward what participants want to see.

**Figure 4:**
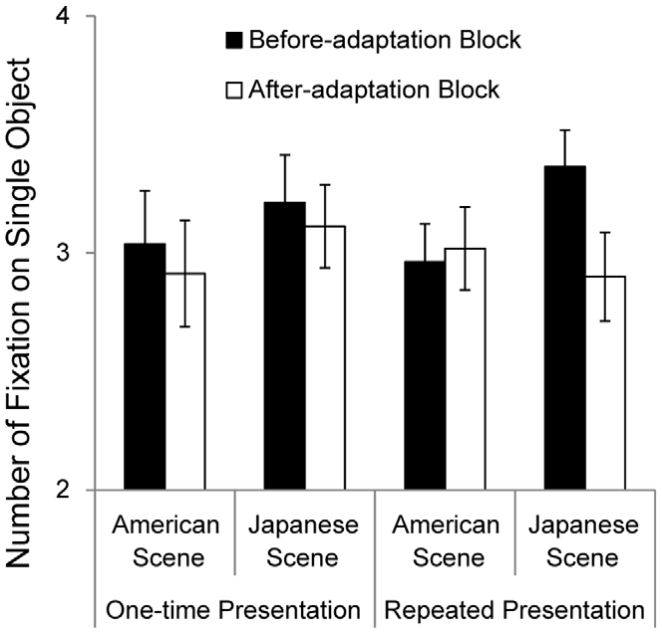
Mean numbers of fixations on the single object during the presentation of single-object photographs. Black bars indicate variances in the before-adaptation block, and white bars indicate variances in the after-adaptation block. Error bars show standard errors.

Next, we investigated the number of fixations on a single object in the same way as in the study by Chua et al. [1]. The mean numbers of fixations on the object under the single-object condition are shown in Figure 4. A 2 (scene: Japanese or American scene) × 2 (block: before or after adaptation) × 2 (presentation: one-time or repeated) repeated-measures ANOVA showed a marginally significant two-way interaction of scene × block × presentation, *F* (1, 30) = 3.10, *p*<.10, *ŋ_p_^2^* = .09. The interaction of scene × block under the repeated-presentation condition was significant, *F* (1, 30) = 5.17, *p*<.05, *ŋ_p_^2^* = .15, whereas it did not reach significance under the single-presentation condition, *F* (1, 30)<1, *n.s*., *ŋ_p_^2^* = .00. The follow-up analyses revealed that participants were less likely to focus on the object after seeing the Japanese scenes than before (3.36 times for the before adaptation block and 2.90 times for the after adaptation block), *F* (1, 15) = 6.22, *p*<.05, *ŋ_p_^2^* = .29, but there was no significant difference between before- and after-adaptation for the American scenes (2.96 times for the before adaptation block and 3.02 times for the after adaptation block), *F* (1, 15)<1, *n.s*., *ŋ_p_^2^* = .01.

## Discussion

This study examined whether the physical environment elicits holistic or analytic attention at the oculomotor level. As the results show, participants were more likely to move their eyes within a broader area when viewing Japanese scenes than they were when viewing American scenes. Moreover, a correspondent pattern was observed even when viewing “culturally neutral” scenes: when viewing single-object scenes, fixations were more likely to be distributed after seeing Japanese scenes than they were after seeing American scenes. Moreover, when participants viewed culturally neutral scenes that they were familiar with, they focused on contextual information more frequently after seeing Japanese scenes than they did after seeing American scenes. This is congruent with Chua et al.’s [1] observation about patterns of eye movements. These results supported our hypothesis that by adapting to a cultural environment, people acquire oculomotor control in accordance with culturally specific styles of attention.

This is the first study to show the influence of the cultural environment on oculomotor control. The Japanese physical environment was more likely to elicit diffused fixations, whereas the American one was more likely to foster object-centered fixations. Note that these observations regarding patterns of eye movements are consistent with those of previous studies that highlighted the dominance of a holistic style of oculomotor control in East Asian cultures and an analytic style of oculomotor control in the U.S. culture [1], [16]. The current results extend those of Miyamoto et al. [13], providing empirical evidence for the presence of common processes of eye movements and their connection with holistic/analytic attention and individual cultural environments. Because of the nature of the physical environment around us, we are always involved in the process of adaptation. Visual attention strategies contribute the most to the emergence of cultural differences.

Further, by revealing the effects of physical environments on eye movements, we posit a new explanation of the inconsistency in the cultural effects of patterns of eye movements in previous studies [1], [17]–[19]. By manipulating exposure scenes, we observed a recalibration process of oculomotor control even with participants from the same culture. This finding suggests that people can rapidly, easily, and flexibly adapt their oculomotor control to new created “cultural” environments. The inconsistency in previous results [1], [17]–[19] might be due to participants’ visual experiences with cultural scenes.

We observed an interaction effect between the top-down (i.e., acquired knowledge) and bottom-up processes (i.e., cultural traits such as complexity) of visual attention. In this study, participants who saw Japanese environments were more likely to distribute their attention while viewing *novel* scenes, but were less likely to focus on a single object when viewing *repeated* scenes. This finding indicates a difference in strategy when one encounters new information. In other words, when participants were placed in novel situations, they were more likely to distribute their attention so as to accumulate as much new information as possible, including information about single objects. In contrast, when the participants were familiar with certain situations because the single object had already been examined, viewing the Japanese scenes (in comparison with viewing the American scenes) prompted them to examine the backgrounds. Despite participants’ possessing prior knowledge of a scene, the physical environment affected specific oculomotor control behaviors.

Although the present study provided empirical evidence for the effects of cultural physical environments on eye movement, several issues remain. One of the most significant limitations is that this experiment was conducted with only Japanese participants. The current results cannot show that factors other than cultural scenes have no effect on the calibration of oculomotor control. To investigate other aspects of culture such as language and emigration, the cultural-scene adaptation paradigm should be utilized along with a culturally diverse sample in the forthcoming study. Moreover, although we assumed that the complexity of cultural scenes leads to the holistic attention style, we could not directly assessed this assumption. Priming with either more complex or less complex scenes, regardless of the places from which the scenes originate, would clarify in more detail the process by which cultural environments affect eye movements.

This study provided empirical evidence supporting the claim that the adaptation of oculomotor control to the cultural environment is linked with cultural styles of visual attention. Rapid adaptation to new “cultural” environments challenges the view held by cultural psychologists that people are deeply immersed in their culture. Culture has many aspects, including ways of communication, language, and landscapes. Further studies will reveal from where cultural differences–and consequently, cultures–originate.
